# Editorial: Bridging the Gap Between the Lab and the Real World: Future Perspectives for Legged Robots

**DOI:** 10.3389/frobt.2020.629002

**Published:** 2020-12-14

**Authors:** Michele Focchi, Daniele Pucci, Andrea Del Prete

**Affiliations:** ^1^Dynamic Legged Systems, Fondazione Istituto Italiano di Tecnologia, Genova, Italy; ^2^Dynamic Interaction Control, Fondazione Istituto Italiano di Tecnologia, Genova, Italy; ^3^Industrial Engineering Department, University of Trento, Trento, Italy

**Keywords:** whole-body control, state-estimation, planning, optimization, perception, model-based control

Legged robots are mainly designed to traverse unstructured environments where wheeled robots have limited mobility due to the presence of obstacles, gaps, stairs, debris. Because of their inherently unstable and hybrid dynamics, legged robots can only drive themselves around by the use of contact forces, which are typically subject to several physical limitations: legs can only push (not pull) on the ground and no arbitrarily large tangential forces can be exerted without slippage. Moreover, the limited capability in terms of actuation and kinematics are additional constraints that further restrict the achievable motions. Sensing a dynamically changing environment is also a challenging task for a robot that has a floating base, because algorithms to estimate its state are required for trajectory planning and control. This topic collects contributions on control, state estimation and planning, which are the main ingredients of legged locomotion, trying to address the open challenges that are still preventing robots to transition from laboratory settings to real-world scenarios.

Xin et al. introduces a computationally efficient controller that achieves a compliant trajectory tracking on the robot. The approach integrates a Cartesian impedance controller with a Quadratic Programming (QP) optimization that relaxes the tracking whenever feasibility constraints are close to violation. In practice, there may not be a physically feasible solution that achieves both the desired motion while satisfying contact constraints independently. A Cartesian impedance control law provides a desired disturbance rejection behavior for the robot base. The impedance is mapped into torques in the motion space taking into account the under-actuation. Then, a single QP is used to find the admissible torques that are as close as possible to the torque commands required for the desired motion performance, trading off performance with feasibility. The approach is faster than state-of-the art whole-body controllers that solve a cascade of QP because it only needs to solve one QP with less decision variables. They also showed experimental validation with the ANYmal quadruped robot walking on a 30-degree slope and on very slippery terrain.

Conversely Raiola et al. proposes a hierarchical whole-body framework able to generate different kinds of gaits, ranging from very dynamic gaits such as the trot, to more static gaits like the crawl. The framework is composed of a priority-based whole-body controller that works in synergy with a walking pattern generator and does not require any higher level planning for the CoM trajectories (i.e., is planner-free), nor state estimation. The approach introduces a smart use of the postural task to remove indeterminacy and achieves consistency between feet and base motion while aiming to keep a well behaved kinematic configuration. In particular, the postural task exploits the redundancy of the higher priority tasks to keep the robot close to a preferable nominal configuration. This generates a connection between the motion of the trunk and the location of the contacts. Therefore, the postural task acts as a set of “elastic linkages” and determines the linear motion of the base, aligning it with the feet. The effectiveness of their locomotion framework was demonstrated on different quadruped platforms such as the Hydraulically-actuated Quadruped (HyQ) and ANYmal.

A framework for state estimation of legged robots, called Pronto, is presented in Camurri et al.. This framework is particularly suited for control purposes, given its ability to provide high-frequency (250–1,000 Hz) state updates—which are fundamental for high-performance state feedback control—based on IMU measurements and leg odometry. Moreover, the estimation can also integrate sporadic low-frequency (1–15 Hz) drift-free measurements, such as those from cameras and LIDARs. These measurements are crucial to ensure that estimation drifts, which are inevitable for IMU and leg odometry, are properly compensated. The reliability and robustness of Pronto was demonstrated with an impressive set of experiments with four different legged platforms: two humanoids and two quadrupeds. This experimental validation is a strong contribution to bridge the gap between research laboratories, where simple motion caption systems can be used to ease the state estimation, and the real world, where complex sensor modalities and update frequencies must be merged in a coherent manner.

A proof-of-concept optimization that improves task feasibility is presented in Lober et al. The paper addresses the well-known sim-to-real problem. This means that there is always a discrepancy between what is planned and what is executed on the real robot, due to modeling errors and perturbations that are not normally captured by model-based controllers. The authors combine a model-based controller and a model-free policy search to efficiently correct initially infeasible motions on real robots. In particular, they define a task feasibility cost function that they optimize in very few iterations using a Bayesian Optimization technique where the minimization of the acquisition function is performed using a gradient-free CMA-ES algorithm. Task trajectories are parametrized via intermediate reference frames, a feasibility cost (energy cost, final goal, etc.) is computed for each roll-out, and finally the policy is optimized with an improved sampling efficiency that balances exploitation with exploration. They compare different experiments on the real robot where the training is bootstrapped or not with simulation data, showing that more aggressive behaviors are produced in the second case. The method is evaluated both in simulation and on the real iCub humanoid robot, showing a sit-to-stand scenario. Because of the sensitive nature of the problem, hand-tuning the trajectory parameters for this scenario would be a difficult chore even for an expert. The result of the optimization is to correct an initially infeasible complex dynamic motion by initially increasing the ground reaction force, and shifting the robot's weight above the heels.

The papers of this topic address several challenges related to the deployment of legged robots in real applications, and therefore represent a step toward bridging the gap between the laboratories and the real world.

## Author Contributions

MF was responsible of editing the paper “An Optimization-Based Locomotion Controller for Quadruped Robots Leveraging Cartesian Impedance Control,” AD was responsible of editing the paper “Pronto: A Multi-Sensor State Estimator for Legged Robots in Real-World Scenarios,” and DP was responsible of editing the paper “Task Feasibility Maximization Using Model-Free Policy Search and Model-Based Whole-Body Control.” All authors contributed to the article and approved the submitted version.

## Conflict of Interest

The authors declare that the research was conducted in the absence of any commercial or financial relationships that could be construed as a potential conflict of interest.

